# Supplementary material for: Sub-nanoliter metabolomics via mass spectrometry to characterize volume-limited samples
Source: Nat Commun. 2020 Nov 6;11:5625. doi: 10.1038/s41467-020-19444-y (PMC7648103; doi:10.1038/s41467-020-19444-y)
Supplement: Supplementary file 1 — Supplementary Information [file 41467_2020_19444_MOESM1_ESM.pdf]

Supplementary Information for

Sub-nanoliter metabolomics *via* mass spectrometry to characterize volume-limited samples.

Yafeng Li[1], Marcos Bouza[1], Changsheng Wu[2], Hengyu Guo[2], Danning Huang[1], Gilad Doron[3], Johnna S. Temenoff [3,4], Arlene A. Stecenko[5], Zhong Lin Wang[2,6], Facundo M. Fernández*[1,4]

[1]*School of Chemistry and Biochemistry, Georgia Institute of Technology, Atlanta, GA 30332, USA.*
[2]*School of Materials Science and Engineering, Georgia Institute of Technology, Atlanta, GA 30332, USA.*
[3]*W.H. Coulter Department of Biomedical Engineering, Georgia Institute of Technology and Emory University, 315 Ferst Drive, Atlanta, GA 30332,*
[4]*Petit Institute for Bioengineering and Bioscience, Georgia Institute of Technology, 315 Ferst Drive, Atlanta, GA 30332*
[5]*Emory+Children's Center for Cystic Fibrosis and Airways Disease Research and Department of Pediatrics, Emory University School of Medicine and Children's Healthcare of Atlanta, Atlanta, GA 30322, USA*
[6]*Beijing Institute of Nanoenergy and Nanosystems, Chinese Academy of Sciences, Beijing 100083, China.*

Corresponding author: Facundo M. Fernández, facundo.fernandez@chemistry.gatech.edu.

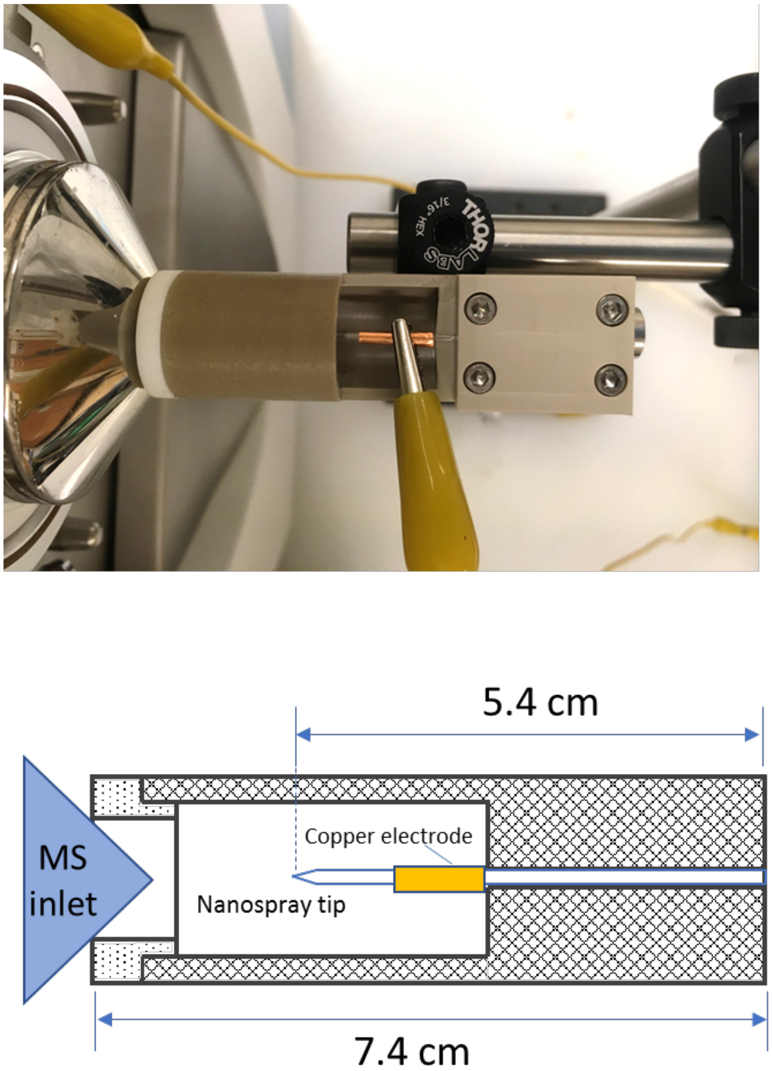

Supplementary Figure 1. Photo and schematic illustration of the TENGi nanoESI mounting cartridge.

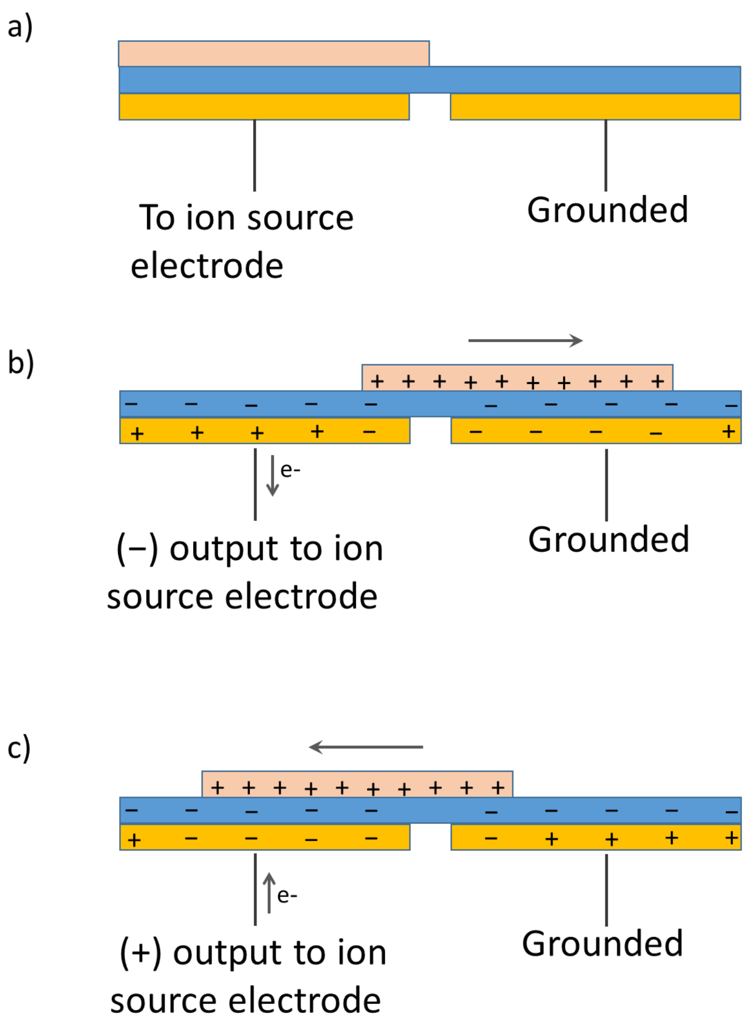

Supplementary Figure 2. TENG charging mechanism illustration. a) With no electrode movement, no charge is generated; b) Sliding the top electrode to the right results in a negative voltage output to the ion source; c) Electrode movement to the left results in a positive voltage output to the ion source.

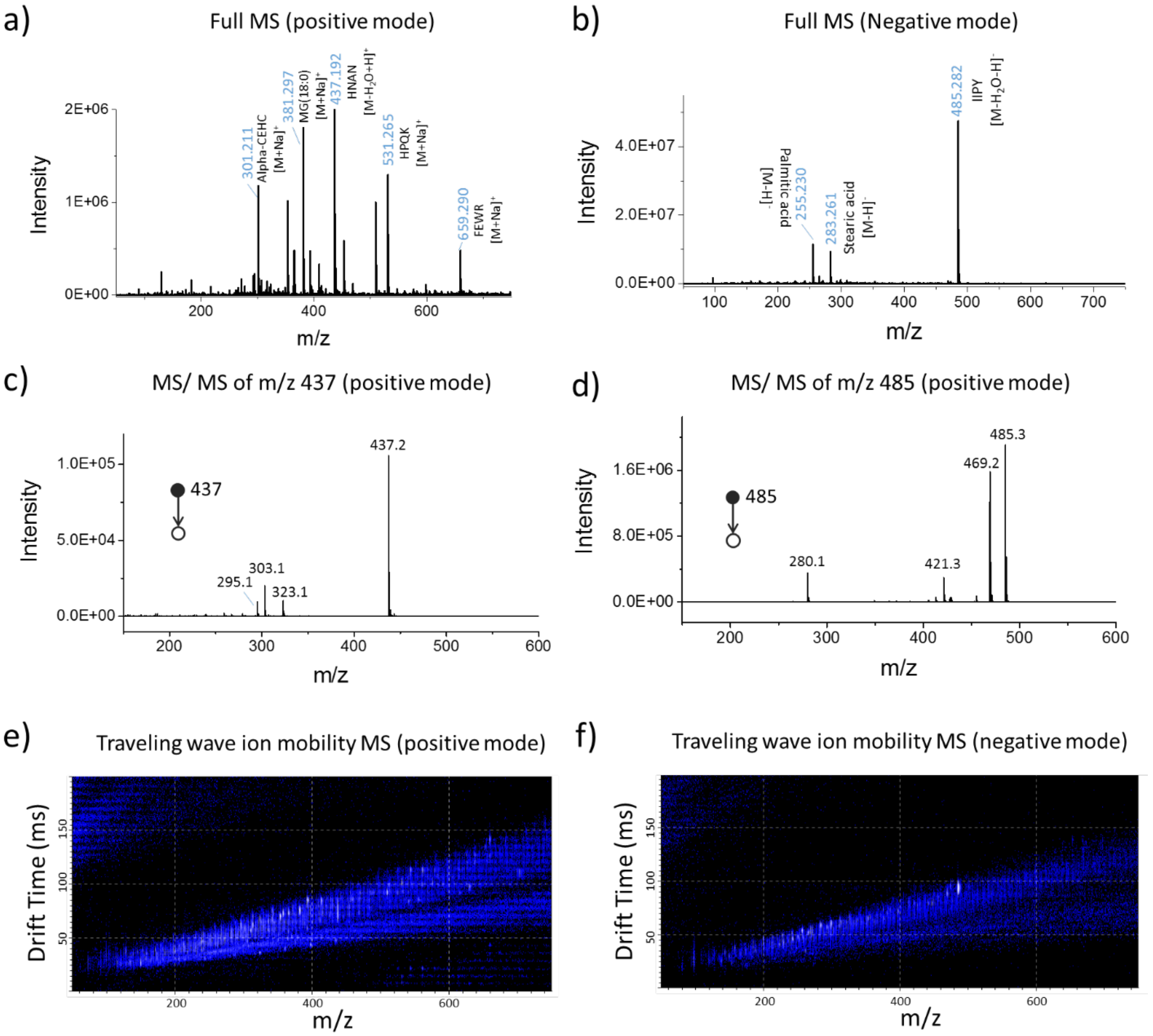

Supplementary Figure 3. TENGi MS analysis of 0.8 µL EBC sample from a healthy volunteer: full MS, MS/MS and ion mobility (IM)-MS in both positive and negative ion mode were completed without the need for loading additional sample. a) Positive ionization mode mass spectrum, after blank subtraction; b) Negative ionization mode mass spectrum, after blank subtraction; c) Tandem MS for the most abundant signal detected in a); d) Tandem MS for the most abundant signal in b); e) Positive ionization mode IM-MS; f) Negative ionization mode IM-MS. In a) and b), peaks labeled are putatively identified as fatty acids (FAs), FA metabolites, peptides, and other human metabolites. (alpha-CEHC indicates alpha-carboxyethyl hydrochroman; species labeled with four capital letters represent tetra-peptides named using standard nomenclature).

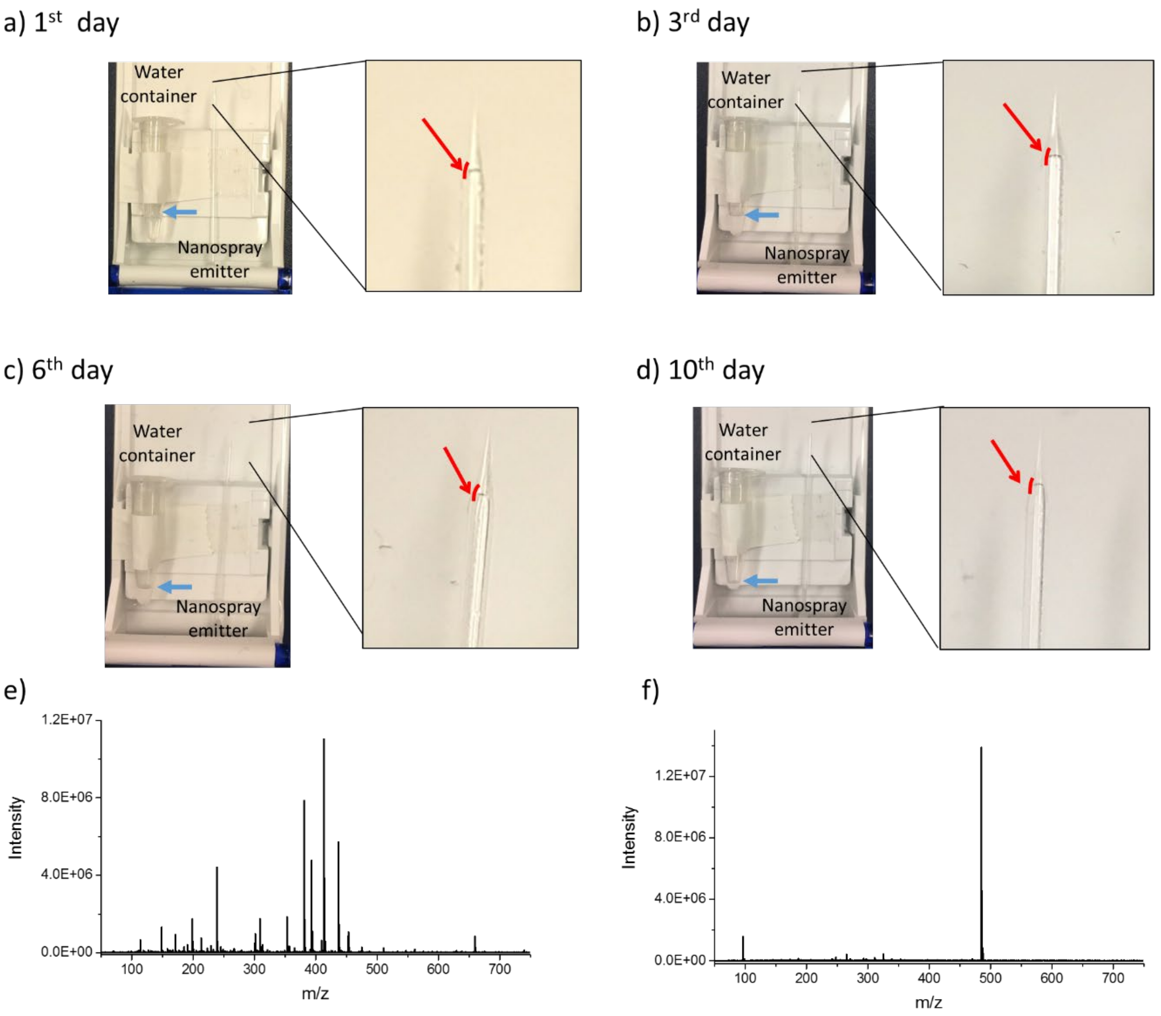

Supplementary Figure 4. Following full MS, MS/MS, and IM-MS experiments (Figure S8), the EBC sample remaining in the nanoESI emitter was refrigerated (4°C) for more than 10 days in a humidified container by sealing the end of the nanoESI emitter with Parafilm (a-d). The water-containing vial depicted in the images had holes drilled on the cap. The blue arrows on the left side of each image indicate the water level in the vial, which decreased over time; the red arrows indicate the amount of sample in the nanoESI emitter, which did not noticeably change over a 10-day period. e-f) blank-subtracted mass spectra in both positive and negative ion modes after 10 days preservation. The relative abundances of the most abundant peaks are similar to those obtained for the fresh samples, shown in Figure S3a and 3b.

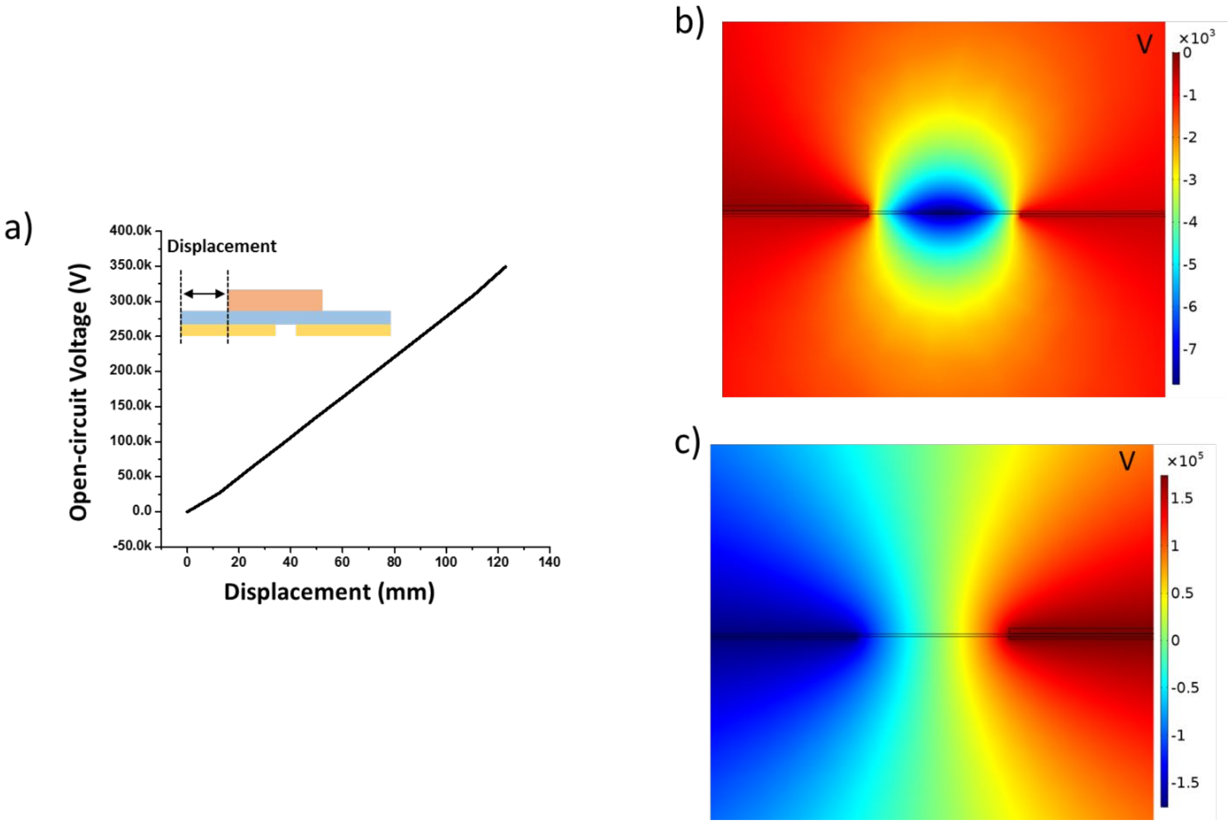

Supplementary Figure 5. COMSOL simulation results of the open-circuit voltage of the TENG device used in this study. a) Relationship between open-circuit voltage and electrode displacement distance. b) and c) voltage distributions for displacement distances of 0 and 120 mm, respectively. With a 0 mm displacement distance, there was no potential difference between the two TENG output electrodes. By increasing the displacement distance, the potential difference increased, reaching values as high as 300 kV when the displacement distance was at its maximum of 120 mm.

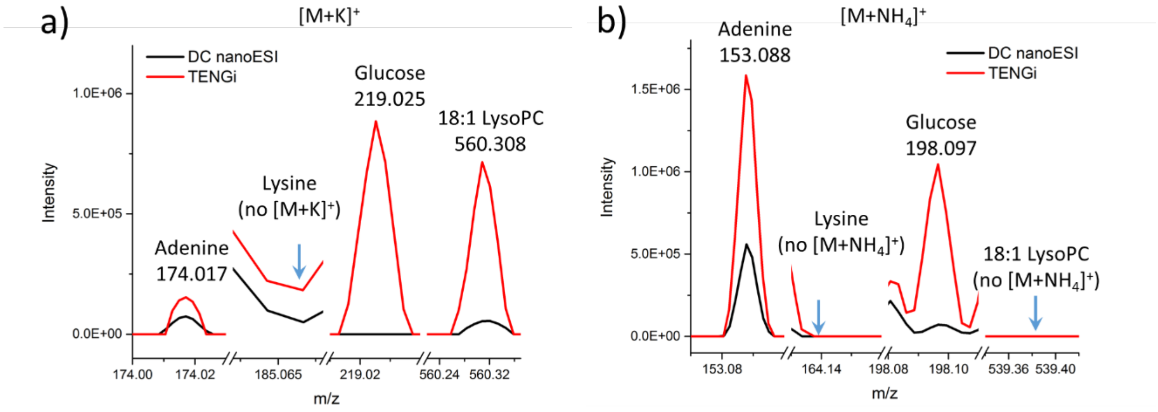

Supplementary Figure 6. Comparison of TENGi and conventional DC nanoESI using four different common metabolites. Additional information of Figure 2d-f.

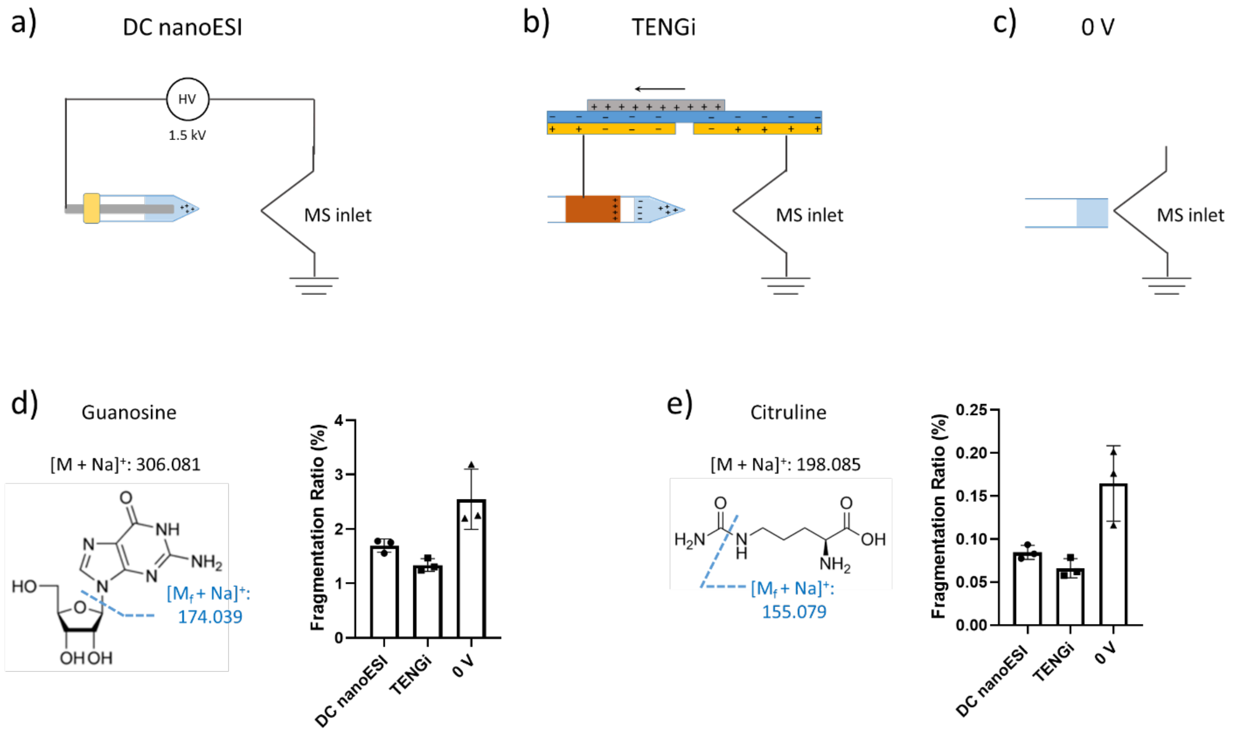

Supplementary Figure 7. Investigation of the extent of in-source fragmentation. Three different ionization methods were compared: a) conventional DC nanoESI (1.5 kV); b) TENGi; c) 0 V inlet ionization. Two metabolites, guanosine and citruline, which have been reported to suffer from in source fragmentation were tested. The fragmentation ratios, calculated as percent of the precursor ion abundance, are shown in d) and e). Data shown are mean value ±SD. Error bars show 95% confidence intervals. n=3 independent experiments.

In-source fragmentation is a confounding factor in metabolomics experiments as many metabolite can yield neutral losses that produce stable species. In-source fragmentation typically occurs in the differentially-pumped regions of the atmospheric pressure interface of the mass spectrometer when voltages are applied that exceed those needed for ion transfer without fragmentation. A number of parameters affect the transport dynamics from the atmospheric pressure source to the vacuum mass analyzer, including those that affect supersonic jet expansion, the intermediate pressure region voltage settings, and the ions own internal energy (*Mass Spectrom. Rev*. 24, 566-587 (2005)).

To assess whether TENGi causes extra in-source fragmentation compared to conventional DC nanoESI, we tested two common metabolites that have been reported to suffer from in-source fragmentation (guanosine and citruline, *Anal. Chem.* 87, 2273-2281 (2015)). Results showed that the in-source fragmentation extent (as measured by the in-source fragmentation ratio) using TENGi was slightly lower than for DC nanoESI, suggesting that although TENG provides much higher voltage than DC nanoESI, no extra in-source fragmentation is induced. This is likely because a) TENG's high voltage is transient and only a limited number of charges are generated per cycle (1.37 µC, Figure 2a) and/or b) is applied indirectly to the sample solution. As a control, the in-source fragmentation ratios for the same two metabolites were also investigated with no ion source voltage was applied. This approach, named 0 V ionization consisted of loading 1 µL of sample into a capillary directly contacting the MS inlet. In this scenario, ionization occurs

through an aerodynamic breakup mechanism as discussed in *Anal. Chem*. 87, 6786-6793 (2015). These experiments showed that fragmentation ratios were higher in the 0V ionization conditions than for both DC nanoESI and TENGi, indicating other factors may play bigger roles in in-source fragmentation than source voltage does.

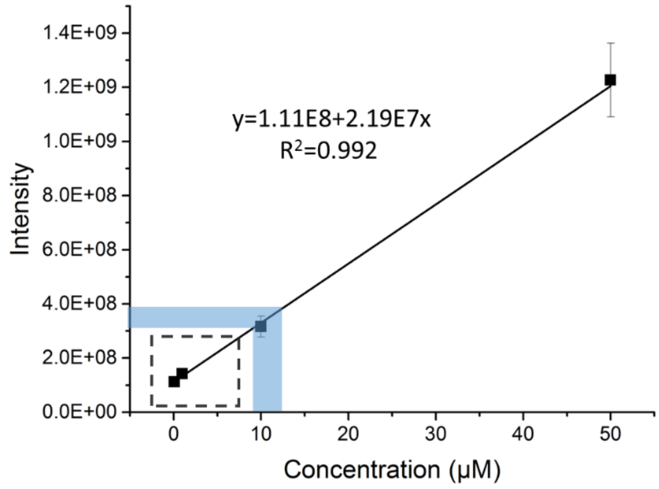

Supplementary Figure 8. Quantification of lactic acid in EBC. Calibration curve was created using lactic acid chemical standard purchased from Sigma-Aldrich. Lactic acid solution (in MeOH:H_2O 1:9) with concentrations of 0.1 µM, 1 µM, 10 µM, and 50 µM were tested 3 times each. Data are mean value ±SD. Error bars show 95% confidence intervals. A healthy volunteer's EBC was collected, concentrated 20-fold and analyzed by TENGi on an Orbitrap QE mass spectrometer. The lactic acid concentration in concentrated EBC was found to be 9.3-12.6 µM (marked blue on the plot, 0.46-0.63 µM in EBC before concentration, n=3 independent repetitions of the TENGi MS analysis).

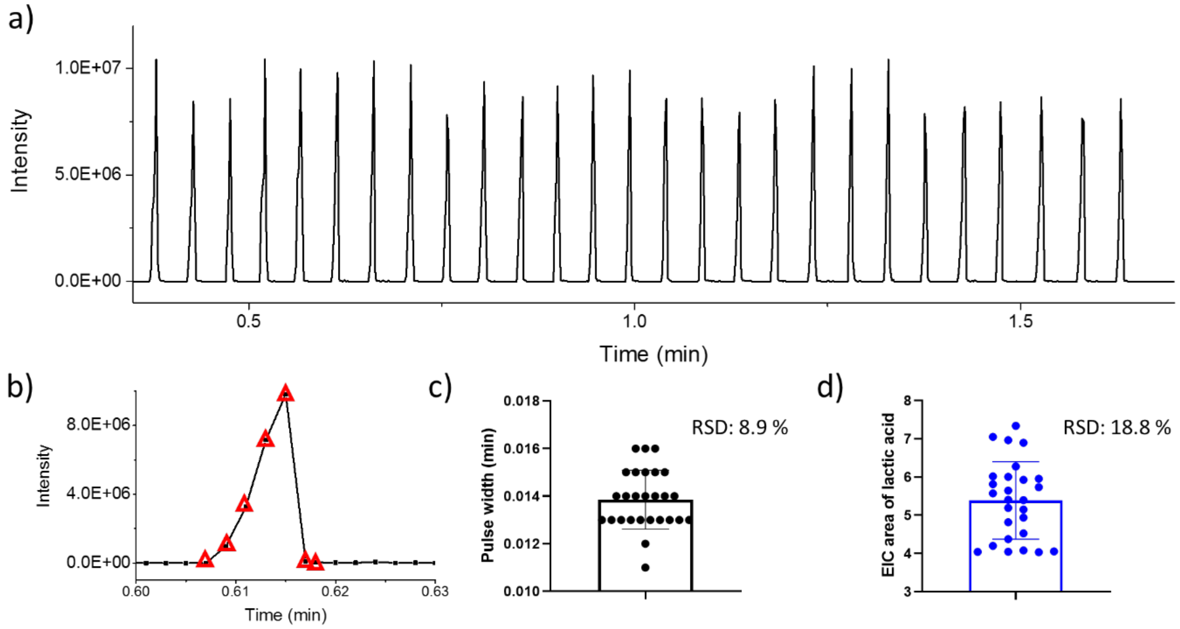

Supplementary Figure 9. Single TENGi pulse characterization. a) TIC of TENGi testing of a concentrated EBC sample from a healthy volunteer. b) the number of scans acquired for one TENGi pulse; c) the RSD of pulse width was 8.9 %, and d) the RSD of the extracted ion chronogram peak area for lactic acid was 18.8%. The first 3-6 pulses generated in a given day of experiments were omitted from all calculations, as the device required these pulses to stabilize the amount of charge delivered. For c) and d), n=27 TENGi pulses shown in a). Data are mean value ±SD. Error bars show 95% confidence intervals.

Supplementary Table 1. Cohort information for cystic fibrosis (CF) patients with impaired glucose tolerance (CF IGT) for which EBC samples were collected and tested by TENGi MS. The last two columns describe if Pre and Post patients successfully yielded EBC samples. OGTT: Oral glucose tolerance test.

Patient No.	Sex	Pre OGTT EBC (Pre)	Post OGTT EBC (Post)
02	Female	Yes	Yes
08	Female	Yes	Yes
09	Male	Yes	Yes
10	Male	Yes	No
11	Male	Yes	Yes
12	Female	Yes	Yes
13	Male	Yes	Yes
14	Male	Yes	Yes
15	Male	No	Yes
16	Male	Yes	Yes
17	Male	Yes	Yes

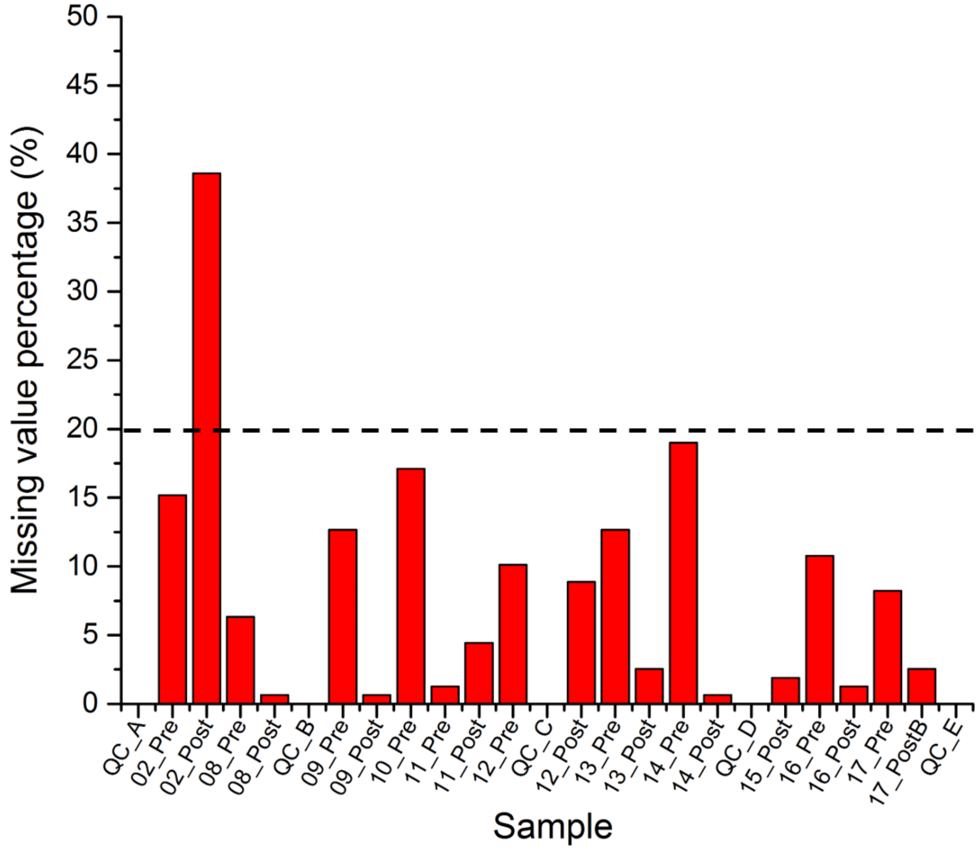

Supplementary Figure 10. Bar chart showing the percentage of missing values for each QC and EBC sample studied.

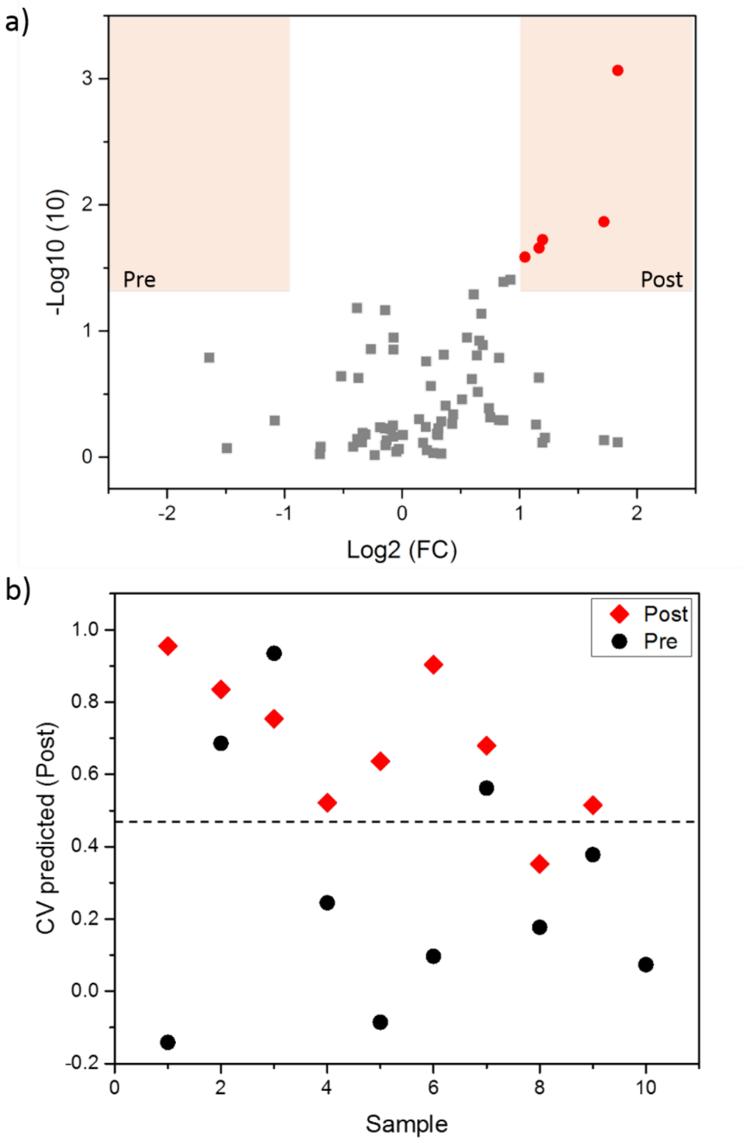

Supplementary Figure 11. a) Volcano plot for features detected in EBC TENGi metabolomics experiments for pre- and post-OGTT CF IGT patients. The 5 red dots denote those features that had p-values <0.05 and fold change (FC)>2. b) oPLS-DA cross-validated classification plot for CF IGT patients pre- and post-OGTT using the 5 most significant features selected in the volcano plot in (a) and PLS-DA VIP scores (Figure 4b). Sensitivity: 89%; specificity: 70%; accuracy: 80%. Sensitivity is defined as the proportion of actual positives that are correctly identified (also known as the true positive rate). In this case, it is calculated as the number of true Post positives samples correctly identified (n=8) divided by the total number of Post samples (n=9). Specificity is the proportion of actual negatives that are correctly identified (also known as the true negative rate). Here it is calculated as the total number of Pre samples that are correctly identified (n=7) divided by the total number of Pre samples (n=10).

Supplementary Table 2. Putative annotation of the five most significant features distinguishing between Pre and Post CF IGT patient groups following TENGi MS experiments. Differences between the two groups were evaluated using the two-tailed student's t test. Tests were calculated on the final dataset after G-log transformation and auto-scaling through MetaboAnalyst. Source data are provided as Source Data file.

m/z	Fold Change (Post/Pre)	p-value	ID	Elemental Formula	Adduct Detected	Mass error (Da)
[a]412.7775	3.5712	0.000861	PS(39:3)	$C_{45}H_{82}NO_{10}P$	$[M-2H]^{2-}$	0.001
[b]318.8149	3.294	0.01362	Trimetaphosphoric acid	$H_3O_9P_3$	$[M+Br]^{-}$	0.003
[a]416.7718	2.2927	0.018889	LacCer(t31:1)	$C_{43}H_{81}NO_{14}$	$[M-2H]^{2-}$	0.0038
[a]372.7842	2.2451	0.022005	[c] (3S)-Hydroxy-tetracosa-6,9,12,15,18,21-all-cis-hexaenoyl-CoA	$C_{45}H_{70}N_7O_{18}P_3S$	$[M-3H]^{3-}$	0.0011
[a]470.7937	2.0674	0.025987	MIPC(d34:0)	$C_{46}H_{90}NO_{16}P$	$[M-2H]^{2-}$	0.0011

[a]Annotated by accurate mass, isotopic distribution (Figure S12) and MS/MS head group fragments (Table S3).
[b]Identified by accurate mass, isotope distribution (Figure S12) and MS/MS fragments (Table S3).
[c]Structure annotation is putative, MS alone does not provide information on isomerism. This metabolite was the only candidate found in the database.

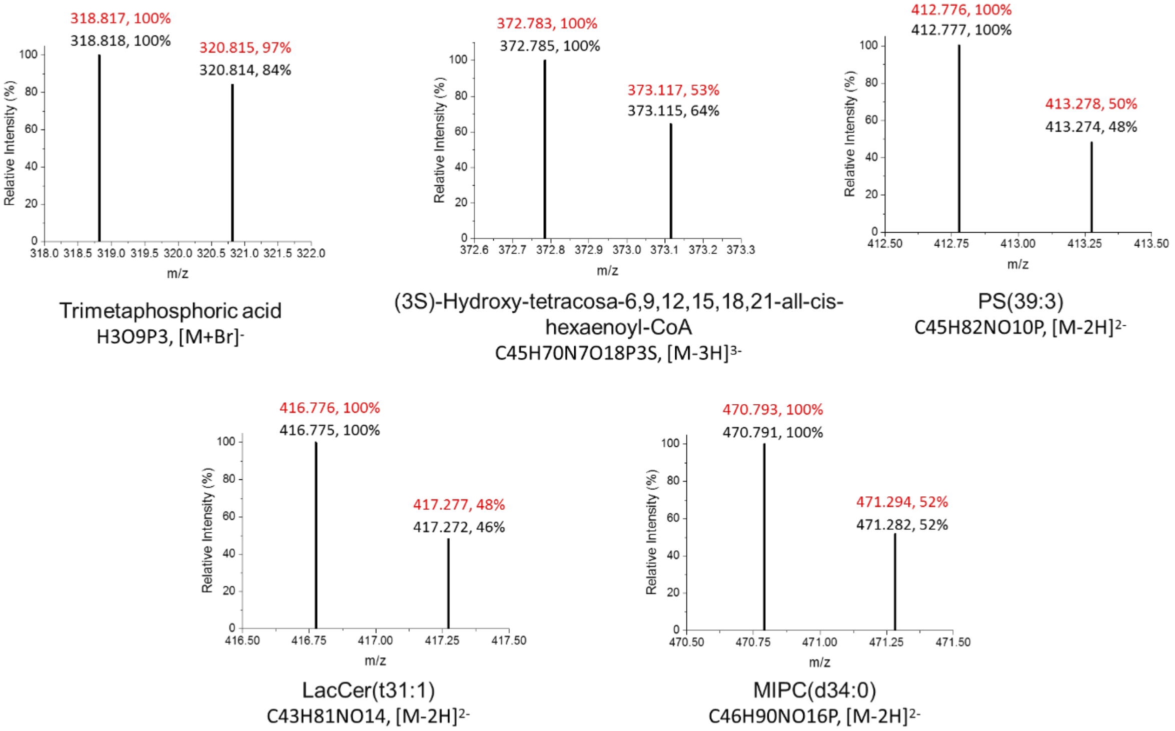

Supplementary Figure 12. Relative isotopic distributions observed by high resolution accurate mass MS for the five most discriminant features detected in EBC from CF IGT patients pre- and post-oral glucose tolerance test. Labels in black correspond to experimental data, while labels in red are for theoretical values.

Supplementary Table 3. Tandem MS fragment matches for the five features that better discriminate EBC from CF IGT patients pre- and post-OGTT.

Feature	Matched fragments (*m/z*, experimental, ∆m⩽0.02)	Proposed fragment ion structure
318.815<br>ID: Trimetaphosphoric acid<br><br>(HMDB: http://www.hmdb.ca/spectra/ms_ms/167318)	78.96	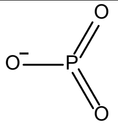
	96.96	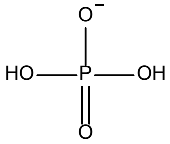
	158.94	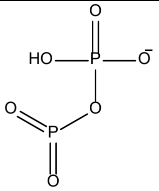
	220.89	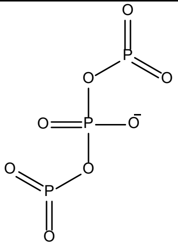
	238.89	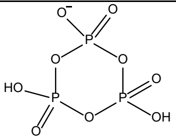
372.784<br>Putative ID: (3S)-Hydroxy-tetracosa-6,9,12,15,18,21-all-cis-hexaenoyl-CoA<br><br>(HMDB: http://www.hmdb.ca/spectra/ms_ms/1372062)	80.96	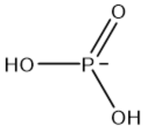
	96.96	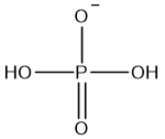
	144.96	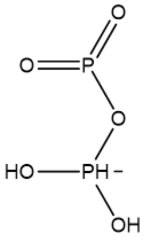

	158.90	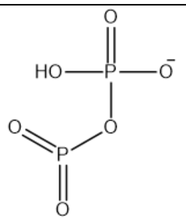
	160.94	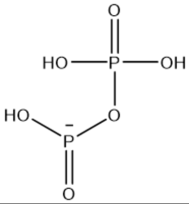
	162.95	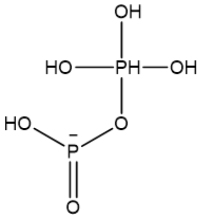
	176.07	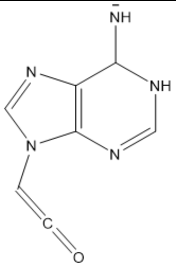
	178.97	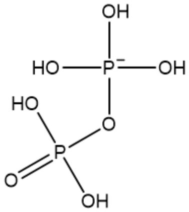
412.778<br>Putative ID: PS(39:3)	80.97	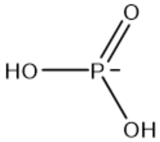
	82.97	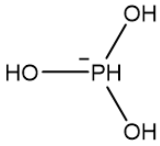
416.772<br>Putative ID: LacCer(t31:1)	147.04	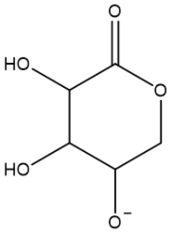

<table>
<tr><td></td><td>221.06</td><td>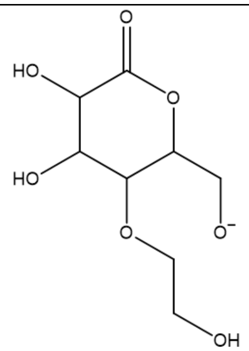
</td></tr>
<tr><td rowspan="3">470.794
Putative ID: MIPC(d34:0)</td><td>96.96</td><td>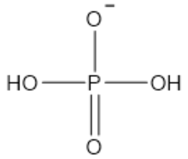
</td></tr>
<tr><td>147.02</td><td>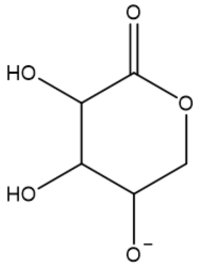
</td></tr>
<tr><td>177.06</td><td>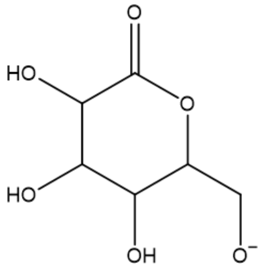
</td></tr>
</table>

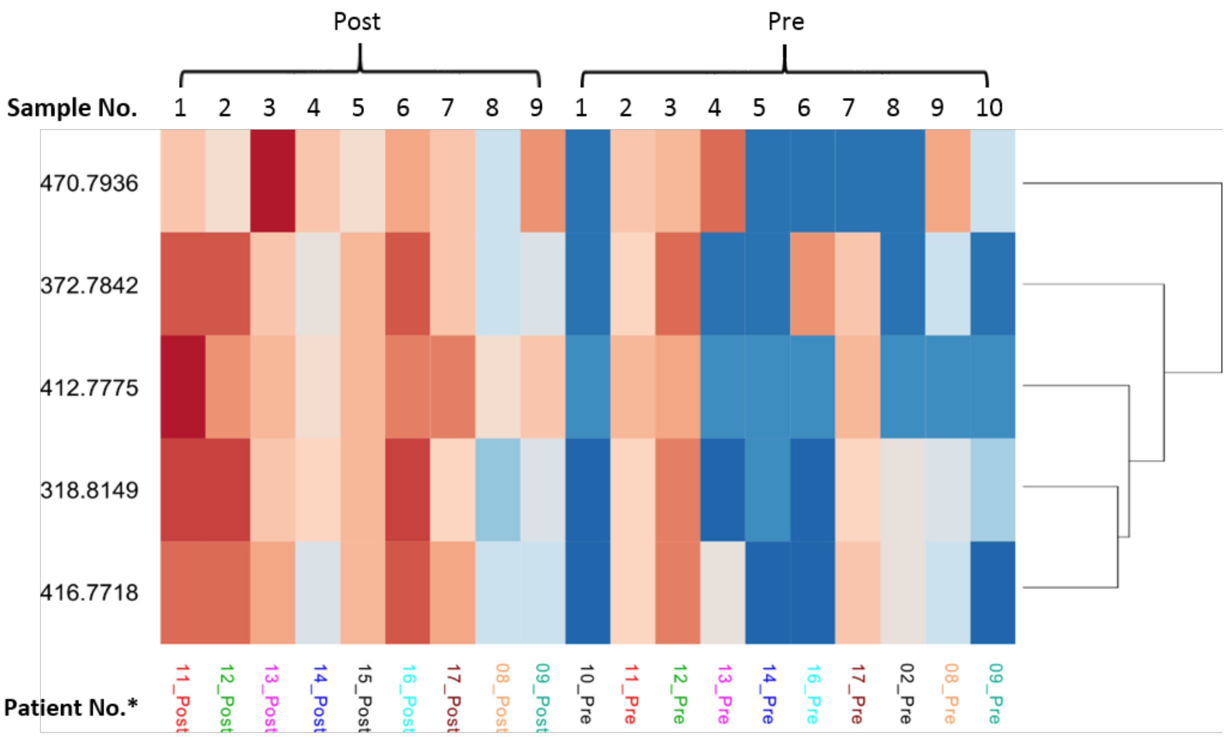

Supplementary Figure 13. Heatmap describing abundances of the 5 topmost discriminant features in EBC (Table S2) from CF IGT patients pre- and post-oral glucose tolerance test.

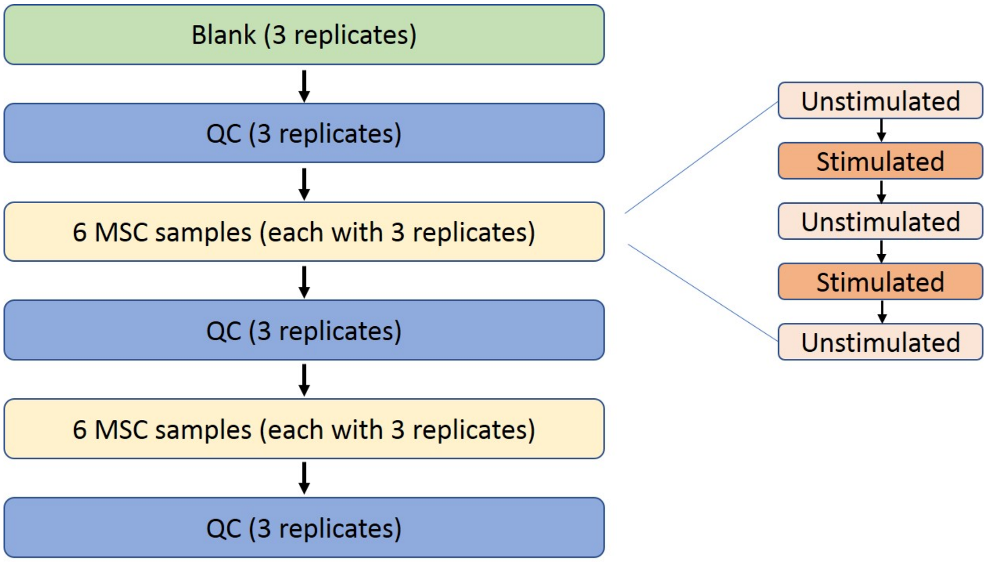

Supplementary Figure 14. Experimental design for TENGi metabolomics of IFN-γ stimulated MSCs.

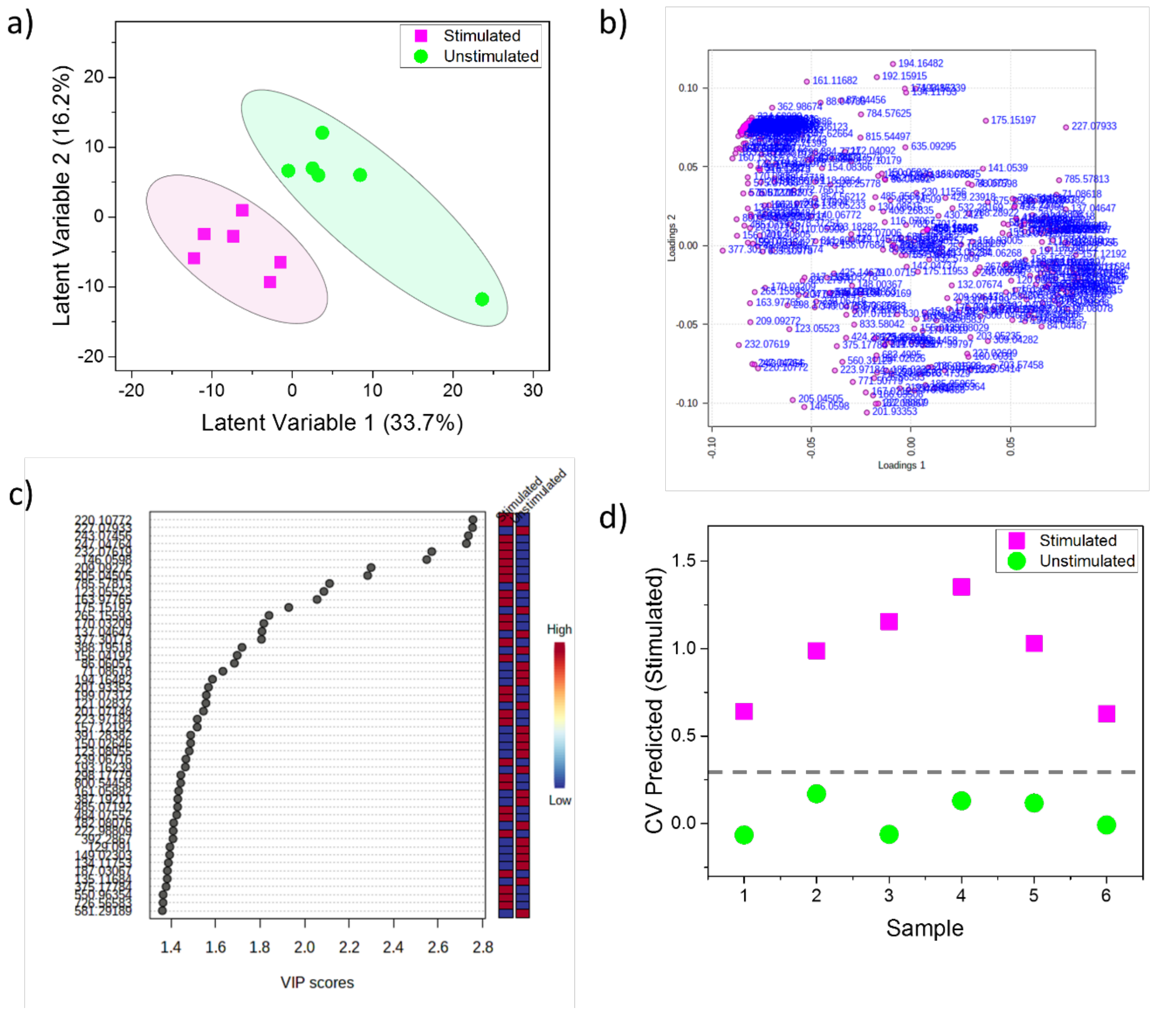

Supplementary Figure 15. Comparison of interferon-γ (IFN-γ) stimulated and unstimulated MSC groups. a) PLS-DA score plot, b) Corresponding loading plot, c) top 50 PLS-DA VIP features; d) oPLS-DA classification plot using the 14 significant feature set. Sensitivity: 100%; specificity: 100%; accuracy: 100%.

Supplementary Table 4. Volcano plot features with p-value<0.05 and FC>2 or FC<0.5. Differences between the two groups were evaluated using the two-tailed student's t test.

m/z	FC	log2 (FC)	p-value	Log10 (p-value)
220.1077	389.23	8.6045	2.45E-15	14.611
227.0793	0.006643	-7.2339	4.63E-14	13.334
243.0746	210.5	7.7177	2.86E-10	9.543
247.0476	256.28	8.0016	1.51E-09	8.8209
232.0762	26.032	4.7022	1.01E-05	4.9939
146.0598	22.202	4.4726	1.76E-05	4.7539
209.0927	15.624	3.9657	0.000763	3.1176
785.5781	0.17973	-2.4761	0.003705	2.4312
123.0552	3.1942	1.6754	0.004415	2.355
163.9777	2.4169	1.2731	0.005411	2.2667
265.1559	2.1676	1.1161	0.017848	1.7484
170.0321	2.3342	1.2229	0.019904	1.701
137.0465	0.14593	-2.7766	0.02067	1.6847
377.3017	4.2472	2.0865	0.020894	1.68
156.0419	2.6953	1.4305	0.033303	1.4775
71.08618	0.16034	-2.6408	0.042675	1.3698

The feature number in the final dataset was 389. Among the 389 features, we tentatively annotated 135 of them based only on their accurate masses (<5ppm). These results are included in Supporting Datasheet-1. Among them are a number of amino acids and their metabolites, purines and their metabolites, lipids etc. As we used positive ion mode for the MSC metabolomics study, most of the TCA cycle metabolites were not detected. But two glycolysis metabolites are found in this list, glucose and fructose 1,6-bisphosphate. These results indicating that the coverage of the TENGi method is wide and able to detect many important metabolites in small population of cells.

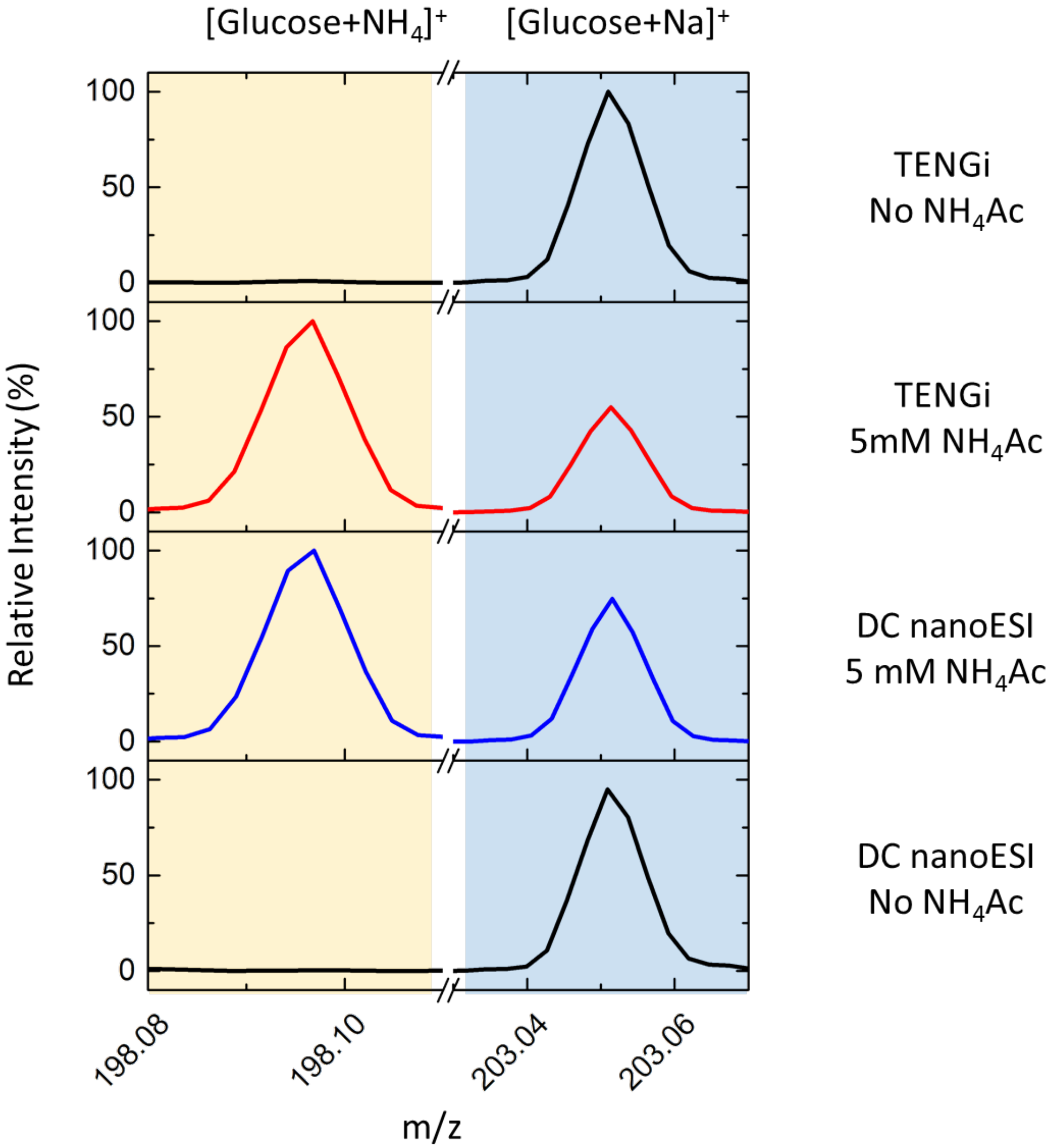

Supplementary Figure 16. TENGi and DC nanoESI MS results for a 10 µM glucose solution with and without 5 mM NH_4Ac addition. Without NH_4Ac addition, glucose was mainly ionized as the [M+Na]+ form with both TENGi and DC nanoESI; with the addition of NH_4Ac the major ion form switched to $[M+NH_4]^+$.

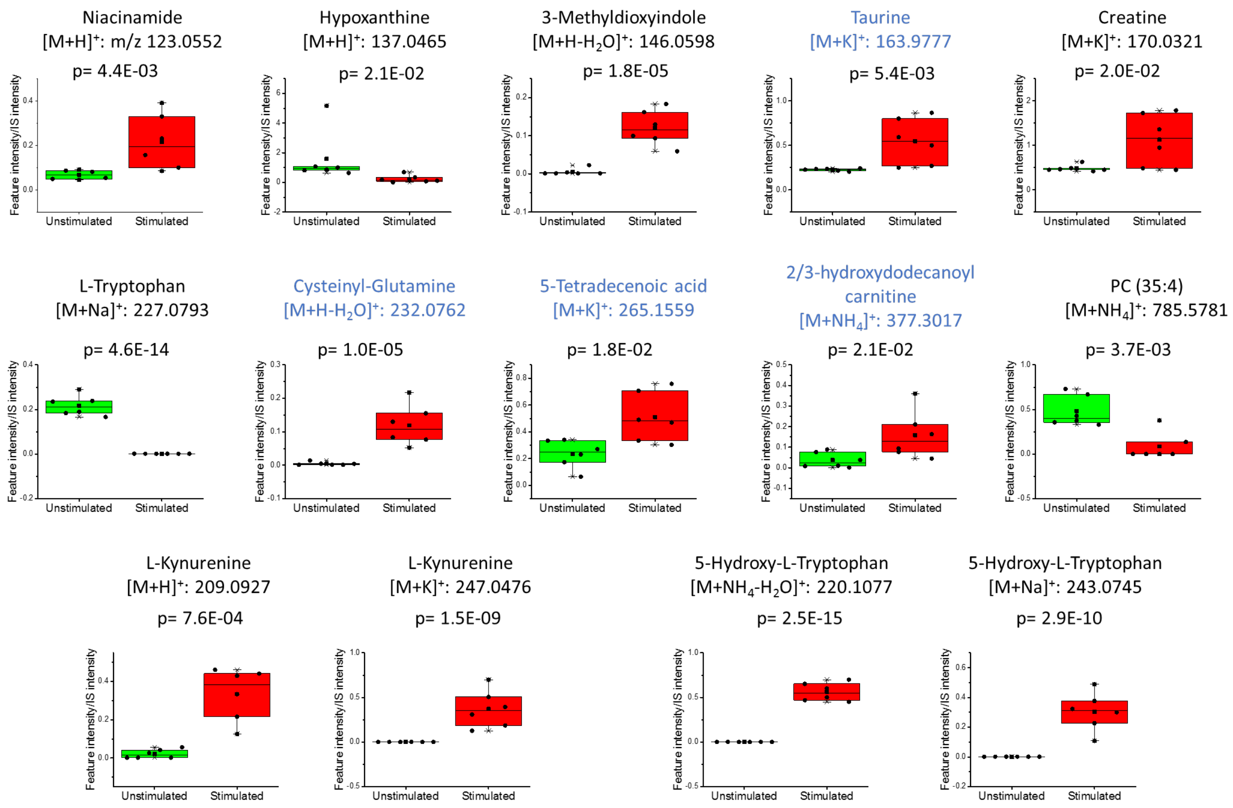

Supplementary Figure 17. Box plots for the 14 most significant metabolites altered in MSCs following IFN-γ stimulation. Metabolites labelled in black were identified by both accurate *m/z* and MS/MS; metabolites labeled in blue have only been putatively identified by accurate mass. Differences between the two groups were evaluated using the two-tailed student's t test (p-value of each feature is shown on each box plot and also provided in Table 1). The lower, middle and upper lines in box plots (c-g) correspond to 25th, 50th, and 75th percentile. The whiskers extend to the most extreme data point within 1.5 interquartile range (IQR).

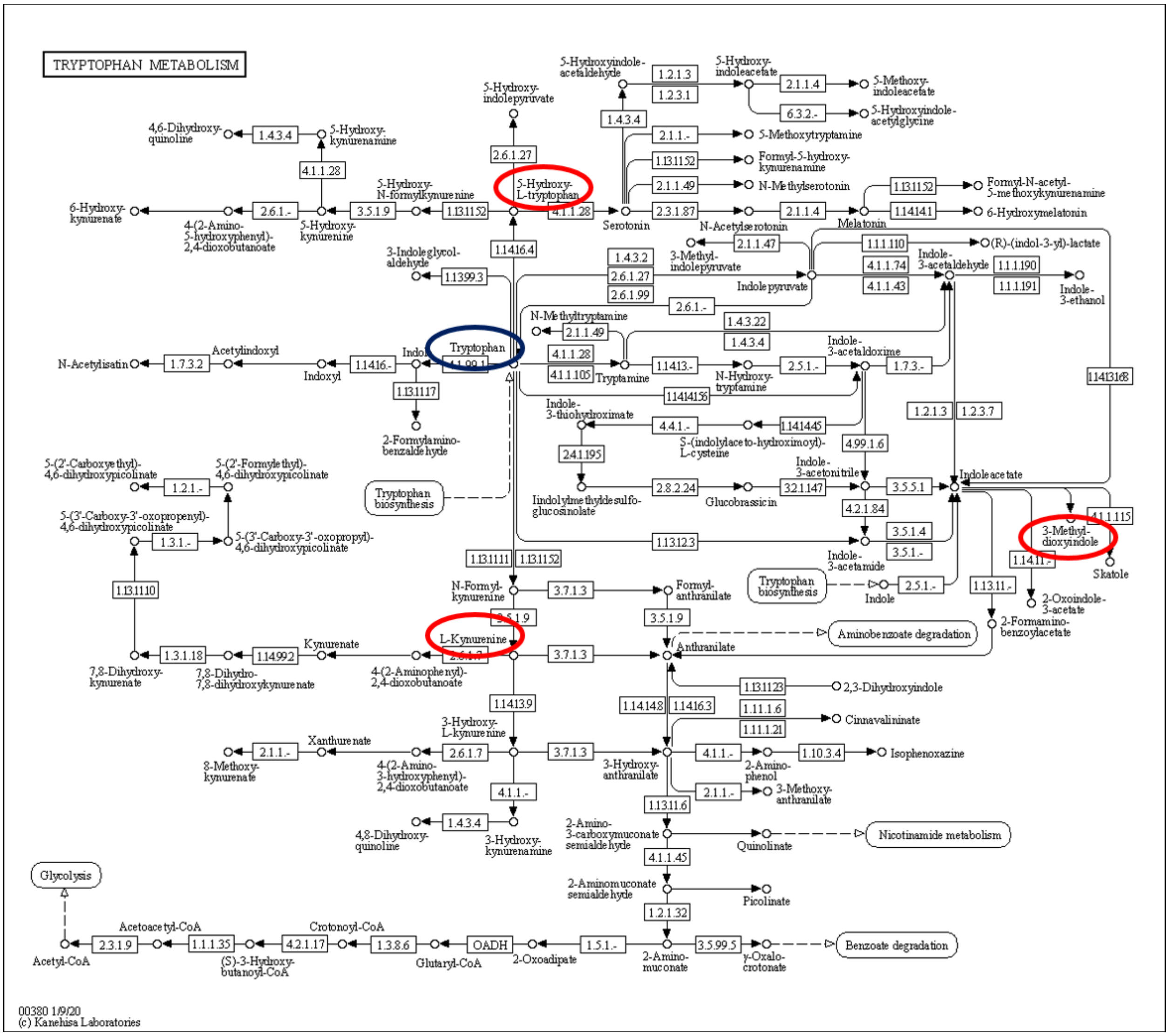

Supplementary Figure 18. Tryptophan metabolism pathway from KEGG (https://www.genome.jp/kegg-bin/show_pathway?map00380). Circled metabolites were detected as significantly altered metabolites upon IFN- γ stimulation of MSCs. The blue circle indicates decrease after stimulation; the red circle indicates increase after stimulation. Additional details are provided in Table 1 in the main text.

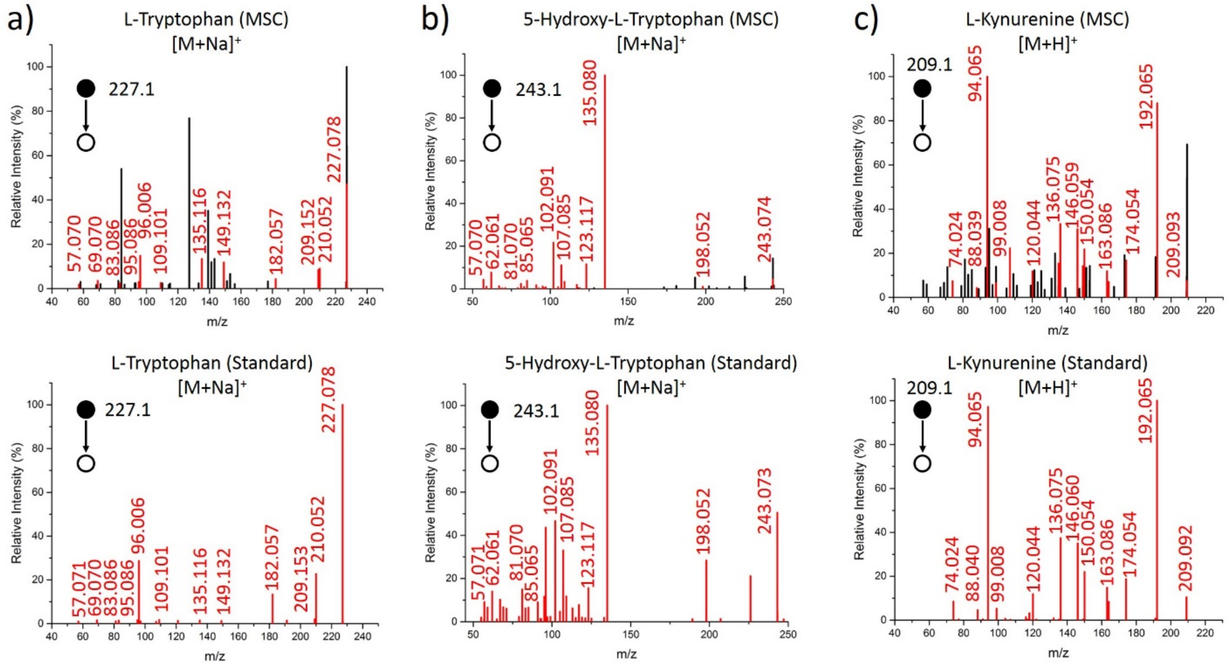

Supplementary Figure 19. Tandem MS (MS/MS) spectra of L-tryptophan (unstimulated MSC), 5-hydroxy-L-tryptophan (stimulated MSC) and L-kynurenine (stimulated MSC) (top row), and from chemical standards (bottom row). Matched fragment ions are shown in red.

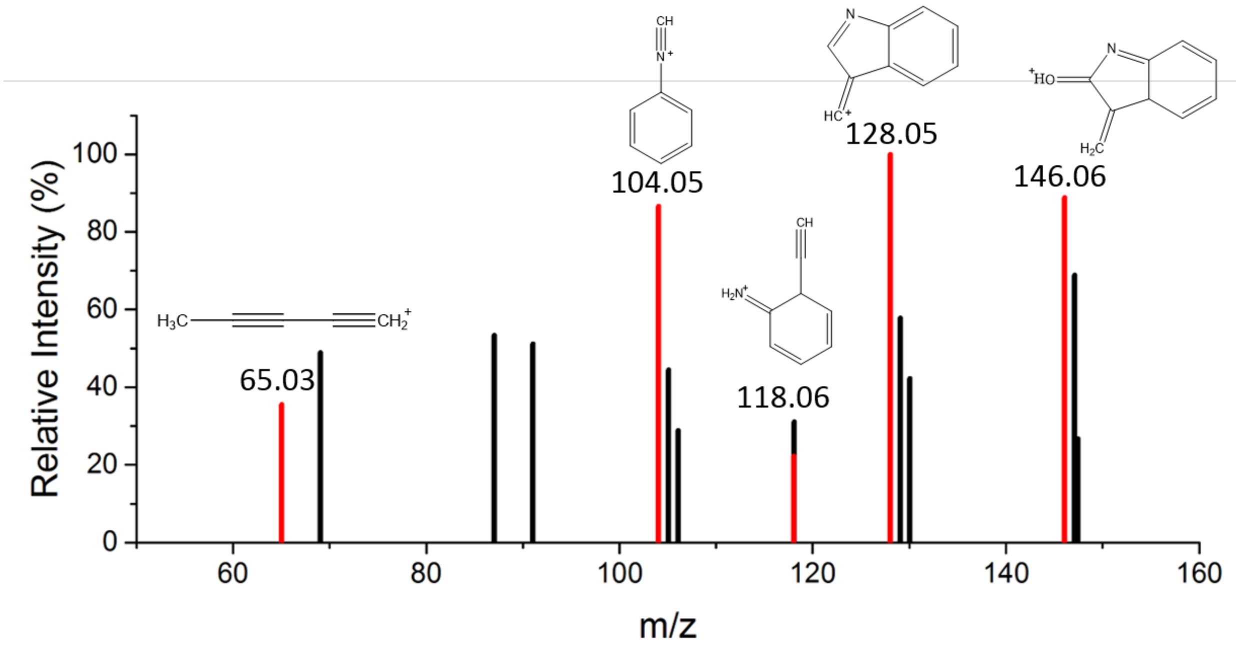

Supplementary Figure 20. Ion mobility-MS/MS (IM-MS/MS) of 3-methyldioxyindole (*m/z* 146.0598, $[M+H-H_2O]^+$). Peaks that are labelled in red correspond to those fragments that matched the *in silico* 40V fragmentation entry in HMDB (http://www.hmdb.ca/spectra/ms_ms/291507).

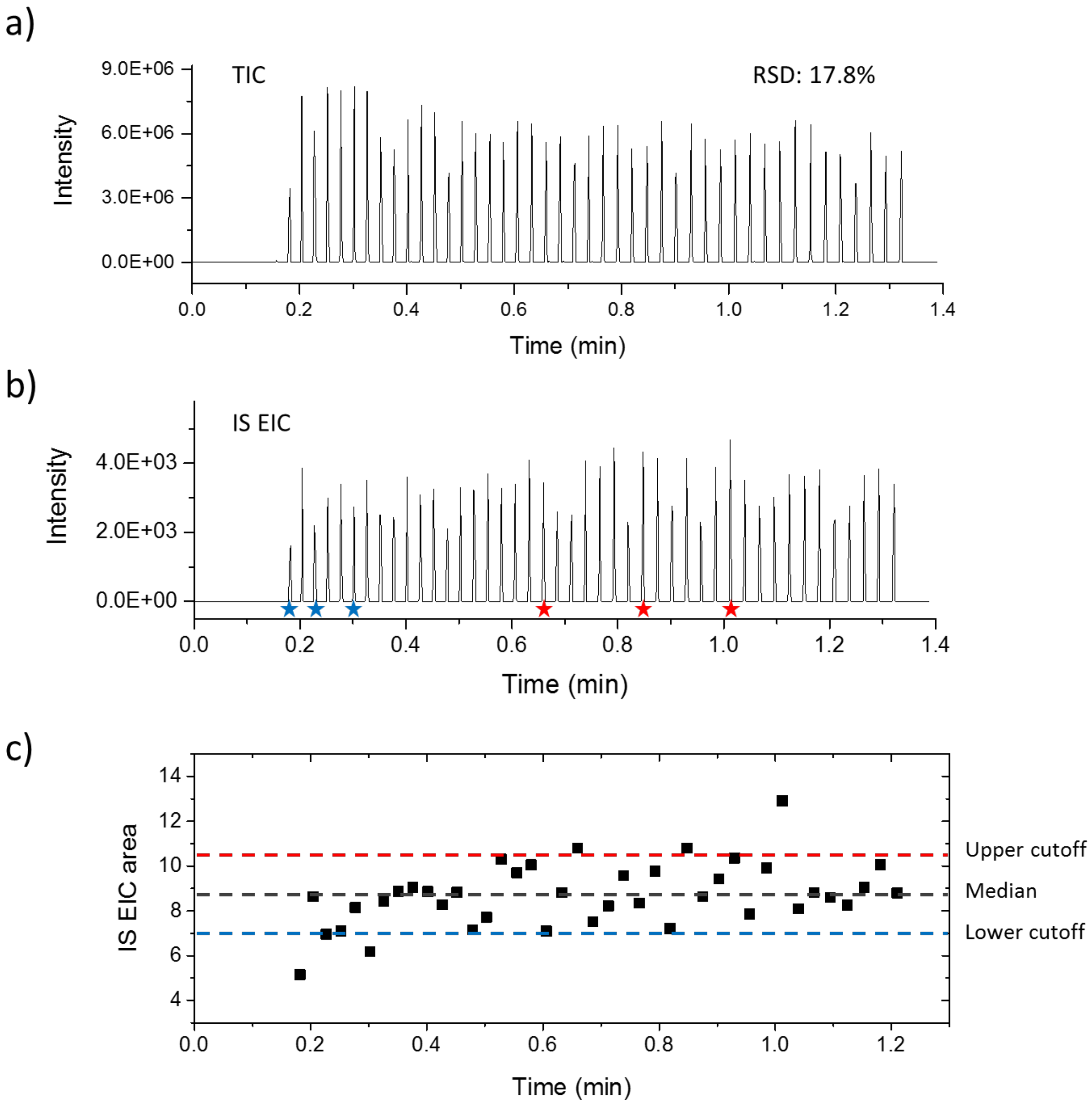

Supplementary Figure 21. Internal standard (IS)-assisted outlier pulse identification. a) TIC for an EBC sample. b) EIC for the 13C tyrosine IS. c) After obtaining the median value for the IS EIC pulse areas, the ±20% interval was calculated. Pulses with IS area below or above this range were deemed outliers. These pulses are marked with stars in b).
